# Is There A Correlation Between COVID-19 and Hepatitis A and Hepatitis E Serum Antibody Level?

**DOI:** 10.30699/ijp.2021.528077.2615

**Published:** 2021-08-02

**Authors:** Alireza Abdollahi, Samaneh Salarvand, Vahid Mehrtash, Bita Jafarzadeh, Reza Ghalehtaki, Saeed Nateghi

**Affiliations:** 1 *Department of Pathology, Imam Khomeini Hospital Complex, Tehran University of Medical Sciences, Tehran, Iran *; 2 *Radiation Oncology Research Center, Cancer Institute, Tehran University of Medical Sciences, Tehran, Iran*; 3 *Department of Cardiology, Tehran University of Medical Sciences, Tehran, Iran*

**Keywords:** COVID-19, Hepatitis A virus, Hepatitis E virus, Polymerase chain reaction, Seroprevalence

## Abstract

**Background & Objective:**

The prevalence of COVID-19 and its severity have been observed to be on a lower level in underdeveloped countries with poorer standards of hygiene. This disparity may be attributed to the higher seroprevalence of other viral diseases, which can result in the presence of antibodies protective against COVID-19. Two of the widespread diseases in such countries are infection to hepatitis A and E viruses (HAV and HEV). In the present study, we explored the relationship between the level of antibodies against these viruses and the susceptibility to COVID-19.

**Methods:**

Ninety patients were studied in two groups of controls and cases, each consisting 45 individuals. The cases were patients with the clinical symptoms of COVID-19 and positive RT-PCR test results. The controls were individuals referred to the respiratory triage of Imam Khomeini Hospital Complex and were not demonstrating relevant clinical symptoms of COVID-19 and their RT-PCR test results were negative. Levels of HAV and HEV antibodies were measured and compared in these two groups.

**Results:**

The median of HAV antibody level was 13.6 (IQR=11.5-16.9) and 13.2 (IQR =10.7-14.7) in cases and controls, respectively, showing no statistically significant difference (*P*=0.1). Likewise, the median of HEV antibody level was 6.7 (IQR=5.3-7.1) and 7.1 (IQR=6.3-7.5) in cases and controls, respectively, which again showed no statistically significant difference (*P*=0.41).

**Conclusion:**

The present study was carried out in a region with a relatively high prevalence of HAV and HEV infections. Contrary to our expectations, no statistically significant relationship was observed between the levels of antibodies against these viruses and the susceptibility to COVID-19. Further studies with larger sample sizes and in other countries are needed to come to a definite conclusion.

## Introduction

It was on March 11, 2020, that the World Health Organization admitted COVID-19 is a pandemic (1). Over the succeeding months, the virus spread almost all countries around the world, though each country had a different pattern of COVID-19-related mortalities and morbidities. Contrary to what is expected based on the healthcare infrastructures of different countries, seemingly countries with lower indicators of socioeconomic development were less severely affected. They had a lower number of reported cases of COVID-19 per million population in comparison to the developed countries (2). Although such variations could be ascribed to the disparity between the availability of diagnostic techniques, financial resources, and access to healthcare services which lead to the underestimation of the actual prevalence, diversity of human pathogens in different geographic regions and concomitant immunological response may also play a protective role (3). One possible explanation for such a difference could be the number of people in a population who have disease-specific antibodies other than the coronavirus antibody, whether acquired through vaccination or previous infections. Viral hepatitis diseases caused by hepatitis A virus (HAV) and hepatitis E virus (HEV), two single-stranded RNA viruses spread by the fecal-oral route, are endemic in countries with low socioeconomic indices (4, 5). The extent of endemicity demonstrated by seroprevalence studies showed a direct correlation of the hygiene and the country’s level of development with the prevalence of infection (6). Iran is one of those countries with a relatively high seroprevalence of HAV and HEV (7, 8). This prevalence offers the opportunity to investigate the association of anti-HAV and -HEV antibodies with the susceptibility to coronavirus infection.

## Material and Methods

This cross-sectional study was conducted on 90 randomly selected patients referred to Imam Khomeini Hospital Complex, Tehran, Iran. Among the hospitalized patients admitted to the infectious diseases ward with the clinical symptoms of COVID-19, 45 cases were selected for the study after performing the Reverse Transcription Polymerase Chain Reaction (RT-PCR) test and confirmation of the infection. Individuals referred to the severe acute respiratory infection (SARI) screening facility of the center who did not meet the criteria of suspected cases of COVID-19 and tested negative for coronavirus infection through RT-PCR were selected for the control group. Individuals with immunodeficiency disorders, end-stage renal diseases, organ transplants, and unstable vital signs were excluded from the study. The patients' chest CT scans were used as a confirmatory diagnostic test. Relevant demographic and clinical data were extracted from the patients' files. 

RT-PCR tests were carried out on naso- and oro-pharyngeal samples which were collected following the WHO guidelines. RT-PCR test was performed on the samples utilizing the Novel Coronavirus (2019-nCOV) Nucleic Acid Diagnostic Kit (PCR-Fluorescence Probing) provided by Sansure Biotech (S3102E) (Changsha, China) which were run on CFX96 Touch Real-Time PCR Detection System (Bio-Rad Laboratories, Inc.) according to the manufacturer’s instructions. Appropriate positive and negative 2019-nCOV-PCR controls were run simultaneously. 

For qualitative determination of total antibodies to HAV (IgM, IgG) and HEV (IgM, IgG, IgA) in the patients' sera, enzyme-linked immunosorbent assay (ELISA) was employed using HEV Ab Version ULTRA and HAV Ab kits provided by Dia.Pro Diagnostic Bioprobes Srl (Sesto, Italy) in accordance with the manufacturer's instructions. Tests were performed on the blood samples, which were taken for biochemical analyses. Test results were interpreted as a ratio of the cut-off value and the optical density measurement at 450 nm (OD 450 nm) using a microplate reader with a 450 nm filter of the sample (Co/S). Co/S values of less than 0.9, 0.9-1.1, and more than 1.1 were interpreted as negative, equivocal, and positive, respectively. The cut-off value was calculated as negative plus positive control test results divided by 3.

The data were analyzed using SPSS software version 26 (SPSS Inc. Chicago, Ill., USA). Data were assessed for normality using the Kolmogorov-Smirnov test. Continuous variables were compared using the independent-samples *t*-test or Mann-Whitney U test where appropriate and reported as the median and inter-quartile range. Categorical variables were reported as frequencies. P-values less than 0.05 were considered statistically significant.

This study was performed after obtaining ethical approval from the Ethics Committee of Tehran University of Medical Sciences, Tehran, Iran.

## Results

In the present study, 90 patients, including 45 cases (23 men and 22 women) and 45 controls (23 men and 22 women), were enrolled. 

From 90 participants, 82 individuals (91.1%) had positive HAV Ab test results with a median of 13.3 (IQR = 11.3-15.7) and 8 individuals (8.9%) had negative HAV Ab test results with a median of 0.42 (IQR = 0.4-0.46). 

Among cases, 43 individuals (95.5%) had positive, and two individuals (4.5%) had negative HAV Ab test results, respectively. Among the controls, 39 individuals (86.6%) had positive and six individuals (13.4%) had negative HAV Ab test results, respectively. The median of HAV Ab levels was 13.6 (Co/S) (IQR=11.5-16.9) and 13.2 (Co/S) (IQR=10.7-14.7) in cases and controls, respectively. The statistical analysis showed no significant difference between the two groups in term of HAV Ab level (*P*=0.1) (Table 1). 

Among all test subjects, 39 (43.3%) and 51 (56.7%) individuals had positive and negative HEV Ab test results, respectively, with a median of 0.5 (interquartile range = 0.18-6.6).

 Among cases, 22 (48.9%) and 23 (51.1%), and among controls, 17 (37.8%) and 28 (62.2%) individuals had positive and negative HEV Ab test results, respectively. The median of HEV Ab level was 6.7 (Co/S) (IQR=5.3-7.1) and 7.1 (Co/S) (IQR=6.3-7.5) in cases and controls, respectively. The statistical analysis showed no significant difference between the two groups regarding the HAV Ab level (*P*=0.41) (Table 1).

**Table 1:** Hepatitis A and E virus antibodies level in COVID-19 patients and uninfected controls

	Coronavirus infected patients (n = 45) Median (IQR)	Control group (n = 45) Median (IQR)	P-value
HAV Ab level*	13.6 (5.4)	13.2 (4)	0.1
HEV Ab level*	6.7 (1.8)	7.1 (1.3)	0.41

* Interpreted as the ratio of the cut-off value and the OD450nm of the sample (Co/S)

## Discussion

This study was conducted to evaluate the effect of preceding HAV and HEV infections on COVID-19 susceptibility. There have been some speculations about the potential protective role of antibodies and immune responses to HAV infections in patients who encountered the novel coronavirus (3). The hypothesis arose when a lower prevalence of coronavirus infection was observed in regions with poor socioeconomic conditions as well as a higher rate of HAV seropositivity in those areas. Despite its seemingly plausible relationship, our study could not detect a statistically significant association between the probability of infection with coronavirus and the level of serum hepatitis A and E antibodies. 

Several pieces of evidence suggest the idea of probable protection against coronavirus infection in the presence of antibodies against viral hepatitis viruses (3, 9). In a review article, Sarialioglu *et al.* illustrated their unpublished observations regarding the low prevalence of coronavirus infection among patients who were under humoral or peritoneal dialysis (around 1 per 1000) compared to normal individuals. They concluded that this could be explained by the high HAV seroprevalence among these patients and pointed to the protective role of HAV antibodies and immune system stimulation following an HAV infection (3). 

The rate of HAV and HEV infections are strongly correlated to the level of access to safe drinking water, socioeconomic status, and hygiene in the region. India, South African countries, and some countries in the Middle East have a high degree of endemicity and seropositivity for HAV and HEV infections. They suffer from poor public health conditions as well (6, 10). Interestingly, when the world encountered the coronavirus pandemic, these countries experienced a slighter increase in COVID-19 infections and a lower mortality rate compared to the developed countries(2, 11). This observation could be explained by a history of previous infections to other viruses which are endemic in those regions and the presence of cross-protective immunity as well as immunization schedules for viral infections. HAV and HEV infections are endemic in the above-mentioned countries, provoking both adaptive humoral and cellular immune responses(12). Furthermore, HAV and HEV vaccines are routinely administered in developing countries, which lead to outstanding immunogenicity, inducing a long-term sustained level of serum antibodies (13, 14).

The COVID-19 course seems to be milder in children, and this age group experiences a lower prevalence of coronavirus infection (15). The presence of antibodies against other viruses that are prevalent in this age group in developing countries could be considered as a reason. Although vaccination for HAV and HEV viruses is not universal in resource-poor countries, it is widely administered for children who are about one year old, leading to the advantageous immunogenicity that induces a high level of immune responses (16-18). Moreover, in developing countries, the vast majority of individuals are exposed to the hepatitis A virus in their childhood, so individuals in this age group have higher anti-hepatitis A virus antibody levels compared to older individuals (19). Therefore, one of the explanations for lower incidence and mortality of COVID-19 in children and young adults could be high levels of circulating antibodies for hepatitis A in these age groups.

The present study was designed to evaluate the potential relationship between the vulnerability to the novel coronavirus infection and the serum levels of hepatitis A and E antibodies. The results of this study that was carried out in one of the countries with a relatively high prevalence of HAV and HEV infections failed to show a statistically significant connection between COVID-19 susceptibility and HAV or HEV antibody levels. 

We believe further studies with larger sample sizes in other geographic regions with higher endemicity of HAV and HEV infections are required before coming to a conclusion. 

## Conclusion

We believe further studies with larger sample sizes in other geographic regions with higher endemicity of HAV and HEV infections are required before coming to a conclusion.

## Conflict of Interest

The authors have no conflicts of interest to declare for this study.
